# Recent epidemiology of patients with gastro-entero-pancreatic neuroendocrine neoplasms (GEP-NEN) in Japan: a population-based study

**DOI:** 10.1186/s12885-020-07581-y

**Published:** 2020-11-14

**Authors:** Toshihiko Masui, Tetsuhide Ito, Izumi Komoto, Shinji Uemoto, JNETS Project Study Group

**Affiliations:** 1 Division of Hepato-Biliary-Pancreatic Surgery and Transplantation, Department of Surgery, Kyoto University, 54 Shogoin Kawaracho, Sakyo, Kyoto, Japan; 2 Japan Neuroendocrine Tumor Society, Kyoto, Japan; 3 School of Nursing at Fukuoka, International University of Health and Welfare, Fukuoka, Japan; 4 Hepato-Biliary-Pancreatic-Neuroendocrine-Tumor Center, Fukuoka Sanno Hospital, Fukuoka, Japan; 5 Department of Surgery, Kansai Electric Power Hospital, Osaka, Japan; 6 Division of Neuroendocrine Tumor Science, Kansai Electric Power Medical Research Institute, Osaka, Japan

**Keywords:** National registry, Neuroendocrine neoplasm, Japan, Incidence, Distribution

## Abstract

**Background:**

The worldwide prevalence and incidence of neuroendocrine neoplasms (NEN) have been increasing recently, although few studies have analyzed data on the current situation of NENs in Japan. Here, the Japan Neuroendocrine Tumor Society (JNETS) planned to investigate the recent incidence and distribution of these tumors using data from the national cancer registry started in 2016. This study examined the incidence and distribution of primary sites as well as rate of advanced disease from this population-based registry.

**Methods:**

A retrospective, population-based study using data from the national cancer registry in Japan (NCR) was conducted to evaluate patients with gastro-entero-pancreatic NEN (GEP-NEN) in 2016. Associated population data were used to determine annual age-adjusted incidences.

**Results:**

A total of 6735 individuals were diagnosed with GEP-NEN in Japan in 2016. Annual onset incidence was 0.70/100,000 for pancreatic NEN and 2.84/100,000 for gastrointestinal NEN. NEN in the ileum accounted for only 1% of total GEP-NENs in Japan. Most NENs in the esophagus or lungs were neuroendocrine carcinomas (NECs), while the majority of those in the duodenum, ileum, appendix and rectum were grade 1 neuroendocrine tumors (NETs). Median age at initial diagnosis was in between 60 to 65. Tumors in the duodenum, appendix and rectum were mostly limited to local, while those in the esophagus, stomach and colon tended to show distant metastasis. In Japan, initial treatment for GEP-NENs was resection even if the tumor was NEC.

**Conclusions:**

This is the first report of a national registry-based incidence and distribution of GEP-NEN in Japan. These data will serve as an important first step to determining the exact etiology and trends for this pathology in Japan.

## Background

Neuroendocrine neoplasm (NEN) was called a carcinoid (carcinoma-like tumor) before and has been considered a rare and indolent tumor [1, 2]. According to the Surveillance, Epidemiology and End Results (SEER) program in the United States, the number of patients with this pathology has been increasing, with an annual incidence of 6.98 per 100,000 population in 2012 [3], *approximately* 7 times greater than the 1.09 per 100,000 population reported in 1973.

In Japan, only two studies have reported on the incidence of NEN. Ito et al. estimated annual incidences in 2005 of 1.01 per 100,000 population for pancreatic NEN (PanNEN), and 2.10 per 100,000 for gastrointestinal NEN (GI-NEN) in Japan [4]. The same group conducted a second survey in 2010 and reported increased incidences of 1.25 per 100,000 for PanNEN and 4.35 per 10,000 for GI-NEN [5]. Although these analyses provided numerous important new insights, such as the low incidence of ileal NEN in Japan, the data were based on questionnaire surveys. A more precise analysis of NEN patients in Japan has thus been necessary to clarify the status quo of NEN diagnosis/treatment in Japan.

In 2016, the Cancer Registry Promotion Act took effect which required all hospitals in Japan to submit the basic data of newly encountered cancer patients to the national cancer registry. Since NEN is rare and displays a comparatively indolent phenotype, these data have not been presented as annual reports. The Cancer Registry Promotion Act recommends utilizing these data for the investigation of cancer epidemiology in Japan, and these data could provide an excellent resource for evaluating the incidence and distribution of NEN in Japan. For accuracy. National Cancer Center asks the facility concerned if they found an inconsistency. Therefore, it takes 3 years to settle the data at present, resulting in analyzing the data in 2016 in this study.

The present study employed 2016 data from the National Cancer Registry to evaluate the number and distribution of primary sites as well as the malignant potential, to elucidate the status quo of NEN in Japan.

## Methods

### Data source

All 2016 submissions to the National Cancer Registry in Japan were used for this study. The Japan NCR is a coordinated system of population-based cancer registries collecting incidence and survival data on cases reported from every hospital in Japan. The pertinent population data are obtained from the Ministry of Health, Labor and Welfare. We collected data from patients with pulmonary and gastro-entero-pancreatic (GEP)-NETs using histologic codes from the International Classification of Diseases for Oncology, 3rd Edition, that identified patients with NENs (8013/3, 8041/3, 8042/3, 8043/3, 8044/3, 8045/3, 8150/3, 8151/3, 8152/3, 8153/3, 8154/3, 8155/3, 8156/3, 8240/3, 8241/3, 8242/3, 8243/3, 8244/3, 8245/3, 8246/3, 8247/3, 8249/3, 8240/3, 8241/3, 8242/3, 8243/3, 8244/4, 8245/3, 8246/3, 8247/3, and 8249/3).

The staging system used for analysis was that adopted by the SEER program. Tumors were classified as localized, regional, or distant. Localized NEN was defined as an invasive neoplasm confined entirely to the organ of origin. Regional NEN was defined as a neoplasm that: 1) extended beyond the limits of the organ of origin directly into surrounding organs or tissue; 2) involved regional lymph nodes; or 3) fulfilled both the aforementioned criteria 1) and 2). Finally, distant NEN was defined as a neoplasm that had spread to parts of the body remote from the primary tumor such as metastasized to the liver, lung, bone and/or distant lymph nodes. In total, analyzed factors included gender, age, primary sites, NETG1, G2 and NEC, stage at diagnosis, methods of discovery and treatment.

The Kyoto University institutional review board approved all study protocols (approval number R2010, September 2019). Data analysis was conducted between December 2019 and February 2020.

### Statistical analysis

NCR histological grade information is designed to be classified according to WHO2010 criteria as: grade (G)1, well-differentiated, Ki67 0–3%; G2, well-differentiated, Ki67 3–20%; neuroendocrine carcinoma (NEC), Ki67 > 20%; or mixed adenoneuroendocrine carcinoma (MANEC), co-localization of NEC with adenocarcinoma.

NENs were classified according to the anatomical location, with the diagnosis of NENs left to the judgment of each institution. Data on disease stage were collected from the final diagnosis at the time of pathological/radiographical analysis. Age-adjusted incidence rates were analyzed using weighted proportions of corresponding age groups according to the 1985 Japan standard population. If the initial treatments included resection, the treatment were dealt as resection, therefore, resection with pharmacotherapy were counted as resection, and pharmacotherapy was counted as pharmacotherapy alone.

All other statistical analyses were performed using JMP version 14.0 (SAS Institute, Cary, NC). Comparative differences were considered significant at *P* < 0.05.

## Results

### Epidemiology of GEP-NEN in Japan in 2016

**Fig. 1:**
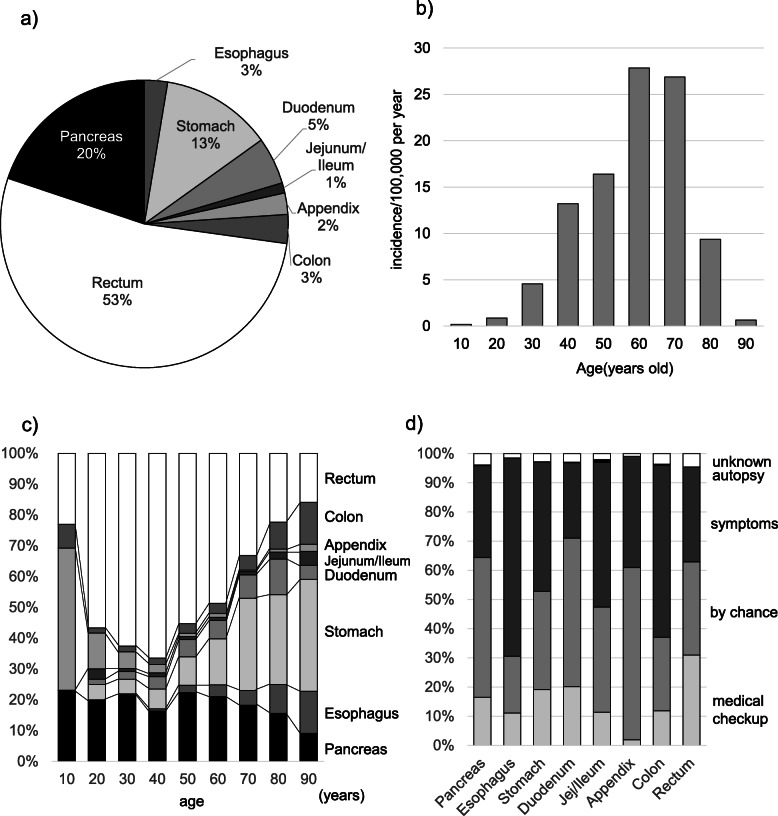
Distribution of primary sites of NENs and age in Japan. **a** Pie chart of GEP-NENs. Rectal NENs comprise 53% of GEP-NENs, followed by the pancreas at 20%, then stomach. **b** Age distributions of GEP-NENs in Japan. The scale is incidence /100,000 per year. **c** Primary site distribution of GEP-NENs for every age decade. **d** Diagnostic opportunity for NENs

Based on data derived from the NCR2016, the total number of patients treated for GEP-NENs in 2016 was recorded as 6735 and the age-adjusted overall incidence was 3.53 per 100,000 population. Age-adjusted incidences of each primary NEN are shown in Table 1 and the ratio is shown in Fig. 1a. About half of GEP-NENs involved the rectum (1.82 per 100,000 population), followed by the pancreas (0.697 per 100,000 population). The incidence of ileal NEN was 1% of total GEP-NENs. For tumor location of PanNEN, 36.0% of all PanNEN patients had the lesion in the pancreatic head, 29.7% in the pancreatic body, and 28.7% in the pancreatic tail.

**Table 1:** Incidence of NEN in Japan in 2016

Primary tumor site	Newly diagnosed number in 2016			Adjusted incidence /100,000 population in 2016		
	Male	Female	All cases	Male	Female	All cases
Pancreas	707	629	1336	0.362	0.335	0.697
Esophagus	200	62	262	0.074	0.025	0.098
Stomach	775	267	1042	0.309	0.120	0.482
Duodenum	264	178	442	0.112	0.083	0.195
Jejunum/Ileum	65	32	97	0.031	0.016	0.046
Appendix	55	50	105	0.039	0.035	0.074
Colon	157	121	278	0.069	0.050	0.118
Rectum	1911	1262	3173	1.099	0.723	1.822
GI-NEN	3427	1972	5399	1.733	1.051	2.835
All cases	4134	2601	6735	2.094	1.385	3.532

Incidence was age-adjusted annual incidence per 100,000 to the standard population in Japan

An apparent male predominance tended to be seen in the incidence of NENs, especially in the upper GI. The overall incidence of NENs among males was 2.09 per 100,000 population and that in females was 1.39 per 100,000 population.

With regard to the age of onset, 65.8% of patients developed NENs in their 60s–70s (Fig. 1b). Age distributions also differed within GEP-NENs according to primary site (Fig. 1c). In the younger patients, the most common primary sites for NEN were the rectum or appendix, and gradually decreased with increasing age. Conversely, the ratio of NENs in the stomach and in the esophagus increased with age. The ratio of NENs in the pancreas was sustained at around 20% in all ages.

The disease was incidentally diagnosed in 51.8% of patients with no symptoms on a visit to the hospital for a health checkup or by chance (Fig. 1d). In pancreatic NEN, only 31.5% of patients showed symptoms on admission, while in GI-NEN, more than half of the esophagus, jejunum/ileum and colon NENs were diagnosed by symptoms.

### Disease grade, stage and metastasis of GEP-NEN in Japan (Fig. 2)

NENs showed various grade distributions for different primary sites. As reported before, most esophageal NENs showed high Ki67 > 20% (WHO2010 NEC), and around half of stomach NENs and colonic NENs were WHO2010 NEC. In contrast, at least 90% of duodenal, ileal, appendiceal and rectal NENs were well-differentiated type, and around 80% of NENs were G1. In the pancreas, at least 79.5% were well-differentiated type and G1 comprised 58.2% of low grade NEN.

**Fig. 2:**
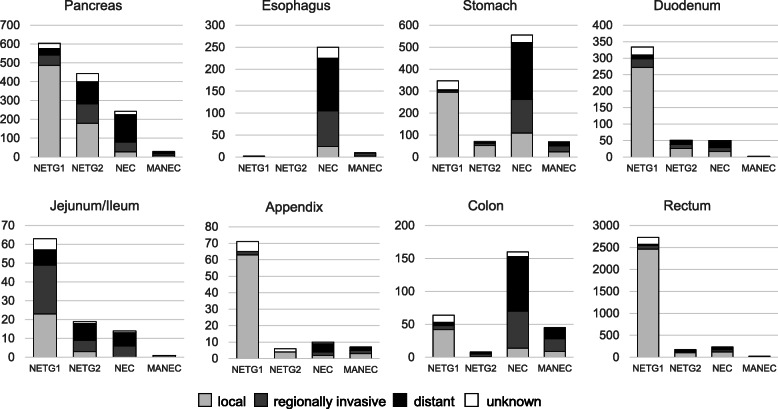
Disease stage of NENs in relation to grade at the time of diagnosis. Faint gray rectangles show localized tumor, gray rectangles depict regional tumor and black rectangles represent tumor with distant metastasis. White rectangles are unknown

Disease stage at the time of diagnosis also differed among primary sites and correlated with higher grade. Esophageal NEN showed distant metastasis in half, while over 70% of duodenal, appendiceal, and rectal NENs remained confined to a local site. In the pancreas, 23.2% exhibited distant metastasis at the time of diagnosis.

### Initial treatment for GEP-NEN in Japan (Fig. 3)

Initial treatment was different among the primary sites of the tumor and the tumor grades. Most GEP-NENs except esophageal NEN were initially treated with endoscopic or surgical resection even if the tumor was WHO2010 NEC. In pancreatic NEN, 29.2% of the WHO2010 NEC were resected initially, although some of the tumors might be diagnosed as NEC in the resected specimens. Because of the tumor characteristics, 24% of esophageal NENs was treated with radiotherapy.

**Fig. 3:**
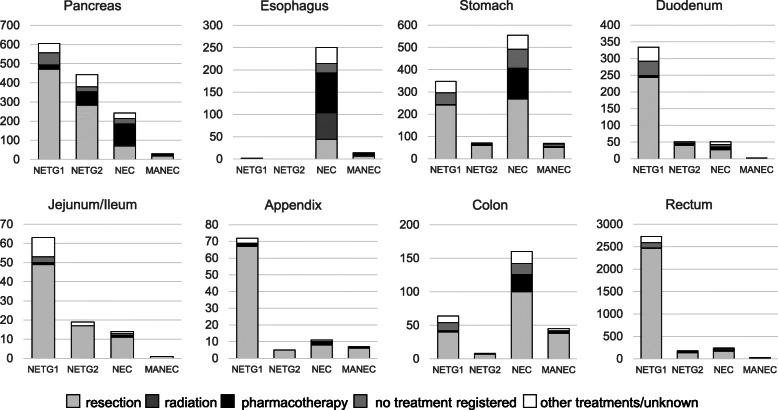
Initial treatment for NENs in relation to grade at the time of diagnosis. Faint gray rectangles represent treatment with tumor resection including endoscopic resection, thick gray rectangles depict treatment with irradiation and black rectangles are pharmacotherapy. Gray rectangles represent no treatment described. White rectangles are other treatments or unknown

## Discussion

This study found that the overall incidence of GEP-NENs in Japan was 3.53 per 100,000 population in 2016. Median age at onset was around the 60s, but patients with rectal NEN were younger than NEN patients with other primary sites. In terms of malignant potential, NENs in the esophagus, stomach and colon were likely to show a more aggressive phenotype, while most NENs in the duodenum, ileum, appendix and rectum were of low grade. Accordingly, distant metastasis was more common with the former NENs, while the latter NENs were likely to remain at a local site.

The number of NEN patients appears to be increasing recently worldwide [3, 6]. In Japan, two reports by Ito et al. in 2005 and 2010 used questionnaire surveys to estimate the incidence of newly diagnosed GEP-NENs as 1.27 per 100,000 population in PanNENs and 3.51 per 100,000 population in GI-NENs in 2010 [4, 5]. Our current estimation was 0.70 per 100,000 population in PanNENs and 2.83 per 100,000 population in GI-NENs, slightly lower than the estimations of Ito et al.

These discrepancies might be attributable to the different methods of estimating incidence, resulting in difficulty in estimating trends for NEN patients in Japan. However, when referring to the age distribution and primary site distribution within NEN patients, median age at onset for individuals with PanNEN and GI-NEN in 2005 was 57.6 years and 59.8 years [5], compared to 65 years for GEP-NEN in the current study. This difference of approximately 5 years could be attributable to the aging of the population in Japan. Indeed, median age in Japan was 42.9 years in 2005, compared to 46.5 years in 2016 according to a population survey by the Statistics Bureau of Japan, representing an increase similar to that seen in NEN patients.

The incidence of GEP-NENs in each age provided three categories according to primary site: 1) decrease group: appendix and rectum; 2) increase group: esophagus, stomach and colon; and 3) steady group: pancreas, ileum and duodenum. Because the increasing group showed a high proportion of NEC (Fig. 2), these different tendencies might imply different mechanisms of development for NENs.

In the 2010 survey, Pancreatic NEC (WHO2010) accounted for 7.5% and GI-NEC for 6.2% [5]. The current analysis revealed that 20.4% of PanNENs and 26.6% of GI-NENs were NEC, including MANEC, suggesting that aggressive phenotypes are more prevalent than expected. Compared to the SEER database as reported by Dasari [3], the overall incidence of GEP-NEC is around 15–20%, comparable to that in our study.

The distribution of primary tumor sites was similar to that in a previous survey from Japan. Half of GEP-NENs were rectal NENs, followed by PanNEN. Comparing to analyses from Europe [7–10], the ileum was much less frequent as a primary site, at 1%. This is consistent with a report from Taiwan [11], which suggested interactions with ethnic and/or regional factors in the development of NENs. Yao et al. [12] presented the incidence of ileal/jejunal NENs among Asian/Pacific Islander and Indian/Alaskan natives as 0.09 per 100,000 population, compared to that in White and African-American individuals as 0.71 and 0.88 per 100,000 population, respectively, suggesting ethnic impacts on the development of NENs.

A tendency toward a male predominance was identified among newly developed GEP-NENs in Japan. The overall incidence among males was 1.73 per 100,000 population, as compared to 1.05 per 100,000 population among females. This is similar to two results using the SEER database in the United States, with 3.65 per 100,000 population among males vs. 3.08 per 100,000 among females in 2000–2004 [12] and males representing 57.75% compared to 42.25% females in 2010–2015 [13].

The current NCR includes data for initial treatment for every patient. As in the Fig. 3, most of the initial treatments for GEP-NEN were surgical or endoscopic resection in every NEN grade even in NEC. It is reasonable that the tumor was resected palliatively or curatively in GI-NEN, because the tumor could cause bowel obstruction. However, even in pancreatic NEC, 29.2% of the tumor were resected. Because 32.9% of the pancreatic NEC was within regionally invasive (Fig. 2), more than 80% of the pancreatic NEC without metastasis were initially resected in Japan. In contrast, treatment of 10.6% of the pancreatic NETG1 was not registered as resection, radiation, chemotherapy, other treatments or unknown. Because NCR registered every element with yes, no or unknown, no treatments above suggest under surveillance or best supportive care. Therefore, for pancreatic NETG1, it is likely that around 10% of the tumor were carefully under surveillance for some reasons in Japan.

This study had several limitations that require consideration when interpreting the results. First, given that NENs may not have been reported to cancer registries unless considered malignant, we have likely underestimated the true incidences and prevalences. This might affect the high incidence of NEC in this registry. Second, the NCR was started in 2016 and we currently lack a system for checking and omitting cases mistakenly registered as newly identified NEN before 2015, implying a potential for overestimation of incidence. In addition, because the registry started at 2016, we could not analyze data except 2016, which may have led to a selective bias. Third, because the NCR was started in 2016, we do not yet have sufficient data on survival. We plan to clarify these issues after we accumulate sufficiently mature data. Finally, several important epidemiological factors are not captured by the NCR. For example, the database does not provide information regarding the hormonal functional status of the NEN, as well as the genetic background. These may affect treatment decisions and survival outcomes that will be analyzed later. Such drawbacks are inherent to any retrospective, population-based study and raise obvious concerns about the generalizability of the findings. However, the size of the present study compensates for this weakness to a great extent and provides a comprehensive epidemiological understanding of NENs in Japan.

## Conclusions

In conclusion, we have provided information on the epidemiological status quo among GEP-NEN patients in Japan by utilizing population-based NCR data for the first time. We plan to expand this study to serial years, and to analyze survivals and trends in mortality afterwards. The current study serves as an important first step to determine the exact etiology and trends in Japan.

## Data Availability

The datasets during and/or analyzed during the current study available from the corresponding author on reasonable request.

## Abbreviations

NEN: Neuroendocrine neoplasm
JNETS: Japan Neuroendocrine Tumor Society
NCR: National Cancer Registry
GEP: Gastroenteropancreatic
NEC: Neuroendocrine carcinoma
NET: Neuroendocrine tumor
SEER: Surveillance, Epidemiology and End Results
GI: Gastrointestinal
MANEC: Mixed adenoneuroendocrine carcinoma
PanNEN: Pancreatic neuroendocrine neoplasm

## References

[1] Berge T, Linell F (1976). Carcinoid tumours. Frequency in a defined population during a 12-year period. Acta Pathol Microbiol ScandA.

[2] Oberg K, Eriksson B (2005). Endocrine tumours of the pancreas. Best practice & research. Clin Gastroenterol.

[3] Dasari A, Shen C, Halperin D (2017). Trends in the Incidence, Prevalence, and Survival Outcomes in Patients With Neuroendocrine Tumors in the United States. JAMA Oncol.

[4] Ito T, Sasano H, Tanaka M (2010). Epidemiological study of gastroenteropancreatic neuroendocrine tumors in Japan. J Gastroenterol.

[5] Ito T, Igarashi H, Nakamura K (2015). Epidemiological trends of pancreatic and gastrointestinal neuroendocrine tumors in Japan: a nationwide survey analysis. J Gastroenterol.

[6] Leoncini E, Boffetta P, Shafir M, Aleksovska K, Boccia S, Rindi G (2017). Increased incidence trend of low-grade and high-grade neuroendocrine neoplasms. Endocrine..

[7] Korse CM, Taal BG, van Velthuysen ML, Visser O (2013). Incidence and survival of neuroendocrine tumours in the Netherlands according to histological grade: experience of two decades of cancer registry. Eur J Cancer.

[8] Scherubl H, Streller B, Stabenow R (2013). Clinically detected gastroenteropancreatic neuroendocrine tumors are on the rise: epidemiological changes in Germany. World J Gastroenterol.

[9] Caldarella A, Crocetti E, Paci E (2011). Distribution, incidence, and prognosis in neuroendocrine tumors: a population based study from a cancer registry. Pathol Oncol Res.

[10] Hauso O, Gustafsson BI, Kidd M (2008). Neuroendocrine tumor epidemiology: contrasting Norway and North America. Cancer.

[11] Tsai HJ, Wu CC, Tsai CR, Lin SF, Chen LT, Chang JS (2013). The epidemiology of neuroendocrine tumors in Taiwan: a nation-wide cancer registry-based study. PLoS One.

[12] Yao JC, Hassan M, Phan A (2008). One hundred years after "carcinoid": epidemiology of and prognostic factors for neuroendocrine tumors in 35,825 cases in the United States. J Clin Oncol.

[13] Tsikitis VL, Wertheim BC, Guerrero MA (2012). Trends of incidence and survival of gastrointestinal neuroendocrine tumors in the United States: a seer analysis. J Cancer.

